# Supplementary material for: The genome sequence of the Small Angle Shades, Euplexia lucipara (Linnaeus, 1758)
Source: Wellcome Open Res. 2023 Nov 9;8:509. [Version 1] doi: 10.12688/wellcomeopenres.20242.1 (PMC11347913; doi:10.12688/wellcomeopenres.20242.1)
Supplement: Peer review history

# Reviewer report: Reviewer response for version 1

**DOI:** 10.21956/wellcomeopenres.22417.r93368

**Published:** 2024-08-29

**Authors:** Vlad Dincă

**Affiliations:** 1 University of Oulu, Oulu, Finland

In this study, the authors have successfully generated a chromosome-level genome assembly of a male *Euplexia lucipara* (Lepidoptera, Noctuidae). The mitochondrial genome has been assembled as well.

 Although I am not an expert in several of the methodological approaches used, as far as I can tell, the manuscript is technically sound and uses methodologies and pipelines that are fairly well-established through use by the Sanger Institute.

 The genome appears to be of high quality and has a BUSCO v5.3.2 completeness of 99.0%.

 It is a pity that the W sex chromosome is lacking (a male was sequenced).

 Reference genomes such as this one represent a valuable resource for the scientific community and each new addition provides new opportunities for research.

**Other comments:**

 I wonder how much is known in terms of genetic structure for *E. lucipara*. Even if limited data is available (e.g. mtDNA, a few nDNA markers etc), if any notable aspect is known (e.g. diverged lineages), it could be worth briefly mentioning it in the Background.

 It may also be worth mentioning whether the species can have more than one generation in other parts of its range.

 Species taxonomy, third line: *Euplexia lucipara* should be written in italics

 Background, first line: “with a wide distribution” or “widely distributed”

Are sufficient details of methods and materials provided to allow replication by others?

Yes

Is the rationale for creating the dataset(s) clearly described?

Yes

Are the datasets clearly presented in a useable and accessible format?

Yes

Are the protocols appropriate and is the work technically sound?

Yes

Reviewer Expertise:

Lepidoptera speciation, taxonomy and phylogeography, with a focus on Western Palearctic butterflies.

I confirm that I have read this submission and believe that I have an appropriate level of expertise to confirm that it is of an acceptable scientific standard.

# Reviewer report: Reviewer response for version 1

**DOI:** 10.21956/wellcomeopenres.22417.r83573

**Published:** 2024-08-26

**Authors:** Jerome H L Hui

**Affiliations:** 1 The Chinese University of Hong Kong, Hong Kong, Hong Kong

Boyes, Lewis, and colleagues report the genome sequence of a male moth *Euplexia lucipara *(Linnaeus, 1758). According to the UKmoths ( www.ukmoths.org.uk), this species is common and widespread over most of the British Isles. Prior to this report, molecular data of this species was rather limited (mainly mitochondrion COI gene sequences deposited to the NCBI database).

 This new genome resource will be useful for further studies such as understanding their population genetic structure, identify cryptic/sub- species, as well as understanding their evolutionary relationships with other moths.

 This genome resource is excellent from the summary statistics, with high BUSCO number, high sequence continuity, and majority of sequences contained on the 30 pseudochromosomes (plus Z chromosome and mitochondrion). To sum up, this is another valuable contribution by the Darwin Tree of Life.

Are sufficient details of methods and materials provided to allow replication by others?

Yes

Is the rationale for creating the dataset(s) clearly described?

Yes

Are the datasets clearly presented in a useable and accessible format?

Yes

Are the protocols appropriate and is the work technically sound?

Yes

Reviewer Expertise:

Genomics, evolution, invertebrates

I confirm that I have read this submission and believe that I have an appropriate level of expertise to confirm that it is of an acceptable scientific standard.

# Reviewer report: Reviewer response for version 1

**DOI:** 10.21956/wellcomeopenres.22417.r87644

**Published:** 2024-07-18

**Authors:** Savarimuthu Ignacimuthu, Sivasankaran Kuppusamy

**Affiliations:** 1 St. Xavier's College, Thirunelveli, TN, India; 2 Entomology Research Institute, Loyola College, Chennai, Tamil Nadu, India

I appreciate the authors for assembling the genome of Small Angle Shades, *Euplexia lucipara* (Linnaeus, 1758).  Authors sequenced approximately 661.6 mega bases using 71 scaffolds. They have used appropriate methods for DNA isolation and used standard software for the sequence assembly and annotations. A total of 20,395 protein-coding genes and 20,590 gene transcripts were observed through the annotations.

**Minor comments on the manuscript**

 The species ( *Euplexia lucipara*) is not in italics in the species taxonomy hierarchy. It can be changed.

 In the background, the second paragraph can be changed as “This is one of a relatively small set of British and Irish insects that feeds frequently on bracken ( *Pteridium aquilinum* (L.) Kuhn) and other ferns (Lawton, 1982).  Its larva also feeds on a wide variety of angiosperms (Henwood et al., 2020). This species has an univoltine in the UK, hibernating in the pupal stage.

 The authors have given the genus name *Euplexia lucipara* full form throughout the article. For the first time the genus name can be given in full form then subsequently can be given in short form like *E. lucipara*

**

 The total length of the mitochondrial genome sequence must be provided in the text.

 Above all, I confirm that the manuscript meets the necessary scientific standards and is suitable for indexing.

Are sufficient details of methods and materials provided to allow replication by others?

Yes

Is the rationale for creating the dataset(s) clearly described?

Yes

Are the datasets clearly presented in a useable and accessible format?

Yes

Are the protocols appropriate and is the work technically sound?

Yes

Reviewer Expertise:

Molecular biology

We confirm that we have read this submission and believe that we have an appropriate level of expertise to confirm that it is of an acceptable scientific standard.
